# Survival After Sentinel Lymph Node Biopsy Compared with Axillary Lymph Node Dissection for Female Patients with T3-4c Breast Cancer

**DOI:** 10.1093/oncolo/oyad038

**Published:** 2023-03-17

**Authors:** Peiyong Li, Ciqiu Yang, Junsheng Zhang, Yitian Chen, Xiaoqi Zhang, Minting Liang, Na Huang, Yilin Chen, Kun Wang

**Affiliations:** Department of Breast Cancer, Guangdong Provincial People’s Hospital (Guangdong Academy of Medical Sciences), Guangdong Medical University, Guangzhou, People’s Republic of China; Department of Breast Cancer, Guangdong Provincial People’s Hospital (Guangdong Academy of Medical Sciences), Southern Medical University, Guangzhou, People’s Republic of China; Department of Breast Oncology, Sun Yat-Sen University Cancer Center, State Key Laboratory of Oncology in South China, Collaborative Innovation Center for Cancer Medicine, Guangzhou, People’s Republic of China; Department of Breast Cancer, Guangdong Provincial People’s Hospital (Guangdong Academy of Medical Sciences), Southern Medical University, Guangzhou, People’s Republic of China; Department of Breast Cancer, Guangdong Provincial People’s Hospital (Guangdong Academy of Medical Sciences), Southern Medical University, Guangzhou, People’s Republic of China; Department of Breast Cancer, Guangdong Provincial People’s Hospital (Guangdong Academy of Medical Sciences), Shantou University Medical College, Guangzhou, People’s Republic of China; Department of Breast Cancer, Guangdong Provincial People’s Hospital (Guangdong Academy of Medical Sciences), Southern Medical University, Guangzhou, People’s Republic of China; Department of Breast Cancer, Guangdong Provincial People’s Hospital (Guangdong Academy of Medical Sciences), South China University of Technology, Guangzhou, People’s Republic of China; Department of Breast Cancer, Guangdong Provincial People’s Hospital (Guangdong Academy of Medical Sciences), Guangdong Medical University, Guangzhou, People’s Republic of China; Department of Breast Cancer, Guangdong Provincial People’s Hospital (Guangdong Academy of Medical Sciences), Southern Medical University, Guangzhou, People’s Republic of China

**Keywords:** breast cancer, sentinel lymph node biopsy, axillary lymph node dissection, survival, seer database

## Abstract

**Background:**

For patients with cN0 and T1-2 breast cancer, sentinel lymph node biopsy (SLNB) can provide survival results equivalent to axillary lymph node dissection (ALND). However, whether it can be performed on T3-4c patients is still controversial.

**Materials and Methods:**

Female patients diagnosed with cN0, T3-4c, and M0 breast cancer from 2004 to 2019 were identified using the surveillance, epidemiology and end results (SEER) database and divided into 2 groups, the SLNB group (1-5 regional lymph nodes examined) and the ALND group (≥10 regional lymph nodes examined). Finally, only those with pN0 disease were included in the SLNB group. The baseline differences in clinicopathological characteristics between groups were eliminated by propensity score matching (PSM). We also conducted subgroup analyses according to age, overall TNM stage, breast cancer subtypes, surgical approaches, radiation therapy, and chemotherapy. The primary endpoint was survival.

**Results:**

With a mean follow-up of 75 months, a total of 186 deaths were reported among 864 patients. The overall survival (OS) and breast cancer-specific survival (BCSS) in the SLNB group were 78.2% and 87.5%, respectively, and that in the ALND group were 78.7% and 87.3%, respectively. The unadjusted hazard ratio (HR) for OS and BCSS in the SLNB group (vs. the ALND group) was 0.922 (95% CI, 0.691-1.230, *P* = .580) and 0.874 (95% CI, 0.600-1.273, *P* = .481), respectively. Besides, the OS and BCSS between the 2 groups were also similar in all subgroup analyses.

**Conclusions:**

SLNB may be performed on female patients with cN0, T3-4c, and M0 breast cancer.

## Teaser

Sentinel lymph node biopsy can provide survival results equivalent to axillary lymph node dissection for patients with cN0 and T1-2 breast cancer; however, whether it can be performed on patients with T3-4c breast cancer is still controversial

**Implications for Practice**

Currently, whether sentinel lymph node biopsy (SLNB) can be performed on female patients with T3-4c breast cancer is unclear. This retrospective study found that the survival after SLNB was similar to axillary lymph node dissection (ALND) among female patients with cN0, T3-4c, and M0 breast cancer when SLNB showed pN0, which means that SLNB may be performed on these patients. In addition, SLNB has significantly fewer complications than ALND, which is important for improving patients’ quality of life. The authors hope that the findings can save these patients from overtreatment (ALND) and help them obtain an improved quality of life.

## Introduction

Sentinel lymph node biopsy (SLNB) is designed to provide survival and regional control results equivalent to axillary lymph node dissection (ALND), but with minimal side effects. Currently, SLNB has replaced ALND as the standard axillary staging surgery for patients with early and cN0 breast cancer. This argument was supported by the National Surgical Adjuvant Breast and Bowel Project (NSABP) B-32 trial, which reported an accuracy of 97.1% and a false negative rate (FNR) of 9.8% for SLNB.^1^ For patients with pN0 disease, we can avoid ALND safely, because the survival after ALND is similar to SLNB.^2-5^ Moreover, there are a lot of complications after ALND, such as arm lymphedema, pain, numbness, weakness, sensory loss, and so on, which limit the patient’s work and daily activities. Fewer complications caused by SLNB compared with ALND can improve patient’s quality of life significantly.^6-9^

However, current studies on SLNB mainly involve patients with T1-2 tumors, for example, more than 98.5% of patients’ T stage in the NSABP B-32 trial were T1-2.^1,2^ Therefore, the American Society of Clinical Oncology (ASCO) guideline does not recommend administering SLNB for patients with T3-4c tumors.^10^ But we should note that the evidence quality of this recommendation is insufficient and we did not find this contraindication in other guidelines, like the National Comprehensive Cancer Network (NCCN) guideline. Although 2 studies had shown that SLNB is feasible in T3/T4b patients with breast cancer, but none of them reported survival results after SLNB.^11,12^

Therefore, to protect female patients with cN0, T3-4c, and M0 breast cancer from unnecessary ALND and its troublesome complications, and gain an improved quality of life, we aim to explore the feasibility of SLNB among these patients by comparing the survival differences between SLNB and ALND.

## Materials and Methods

### SEER Database and Case Selection

All data were extracted from the surveillance, epidemiology, and end results (SEER) 17 registries database released in April 2022 using the SEER*Stat software (version 8.4.0). SEER, an authoritative source for cancer statistics in the U.S., currently collects and publishes cancer incidence and survival data from population-based cancer registries covering approximately 48.0% of the U.S. population. It contains information about patient’s demographics and cancer characteristics, such as sex, age and year at diagnosis, race, marital status, grading, staging and histological types of tumor, surgical approaches, radiation therapy and chemotherapy, survival time, cause of death, and follow-up.

**Figure 1.**
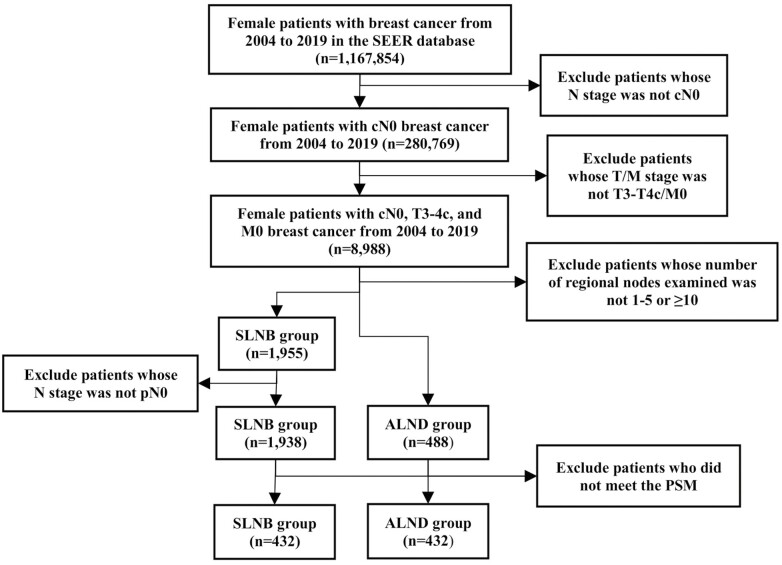
Flow diagram for selection of patients. Abbreviations: ALND, axillary lymph node dissection; PSM propensity score matching, SEER, surveillance, epidemiology, and end results; SLNB, sentinel lymph node biopsy.

The selection process of patients is shown in Fig. 1. First, we identified 1 167 854 female patients diagnosed with breast cancer from 2004 to 2019 using the SEER database. The inclusion criteria were female patients with cN0, T3-4c, and M0 breast cancer, who underwent SLNB or ALND. They were divided into 2 groups, the SLNB group, and the ALND group. Since the SEER database does not specify the type of axillary surgeries, therefore, after referring to previous studies using the SEER database and the NCCN guideline (at least 10 lymph nodes removed are required for ALND to accurately stage the axilla),^13^ our classification of patients into the SLNB or ALND groups relied on the following 3 assumptions: (1) patients with a definite number of lymph nodes examined underwent SLNB or ALND using the codes of “regional lymph nodes examined” (SEER Registrar Staging Assistant, Collaborative Stage Data Collection System version 02.05;^14^ (2) most patients with ≥10 lymph nodes examined underwent ALND;^15,16^ (3) most patients with 1-5 lymph nodes examined underwent SLNB alone.^16-18^ Another reason why we set the upper limit of SLNB at 5 is that resection of 5 lymph nodes can detect more than 99% of positive sentinel lymph nodes^19^ and result in an FNR of 1%.^1^ In conclusion, we assigned patients with 1-5 lymph nodes examined to the SLNB group and patients with ≥10 lymph nodes examined to the ALND group. Finally, only those with pN0 disease were included in the SLNB group, while the ALND group included patients with all pathological lymph node statuses. Propensity score matching (PSM) was used to eliminate the baseline differences in clinicopathological characteristics and survival outcomes between groups. In the end, each group consisted of 432 patients.

### Data and Statistical Analysis

We not only compared the OS and BCSS between the SLNB group and the ALND group in the whole study cohort but also conducted subgroup analyses according to age, overall TNM stage, breast cancer subtypes, surgical approaches, radiation therapy, and chemotherapy. In addition, we compared the effects of different molecular subtypes on survival in all patients in the SLNB group who had received chemotherapy. OS was defined as the period from being diagnosed with breast cancer to death from any cause, and BCSS was defined as the period from being diagnosed with breast cancer to death from breast cancer. The Chi-square test was used to compare the differences in clinicopathological characteristics between the 2 groups, the Kaplan-Meier method was used to evaluate OS and BCSS, and the log-rank test was used to calculate the *P* value (*P* < .05 was considered to be statistically different), as for the HR and 95% CI, they were calculated by the Cox proportional hazard model. The Statistical Product and Service Solutions (SPSS) (version 26.0) was the software used for statistical analysis.

## Results

### Patient’s Characteristics in Our Study Cohort

The clinicopathological characteristics of patients in our study cohort before and after PSM were summarized in Tables 1 and 2 respectively. As shown in Table 1, 79.9% (1938 of 2426) of patients underwent SLNB, while only 20.1% underwent ALND. Most patients were older than or equal to 50 years old (68.6%), were White people (74.4%) and in stage II (73.3%), had undergone mastectomy (64.9%), had received radiation therapy (53.1%) and chemotherapy (75.5%), and the most common breast cancer subtype was HR+/HER2− (luminal A) (39.2%). Table 2 indicates that both groups contained 432 patients with similar clinicopathological characteristics after PSM.

**Table 1.** Comparison of clinicopathological characteristics between sentinel lymph node biopsy (SLNB) and axillary lymph node dissection (ALND) groups in unmatched patients.

Characteristics	Patients, no. (%)			*P*-value
	All patients *N* = 2426	SLNB group *N* = 1938 (79.9)	ALND group *N* = 488 (20.1)	
Age, years				.002
<50	756(31.2)	576(29.7)	180(36.9)	
≥50	1670(68.6)	1362(70.3)	308(63.1)	
Race				<.001
White	1805(74.4)	1,473(76.0)	332(68.0)	
Black	331(13.6)	232(12.0)	99(20.3)	
Others/unknown	290(12.0)	233(12.0)	57(11.7)	
T				<.001
T3	1980(81.6)	1617(83.4)	363(74.4)	
T4a-c	446(18.4)	321(16.6)	125(25.6)	
N				<.001
pN0	2410(99.3)	1938(100.0)	472(96.7)	
pN+	16(0.7)	0(0.0)	16(3.3)	
Stage				.002
I	34(1.4)	31(1.6)	3(0.6)	
II	1778(73.3)	1443(74.5)	335(68.6)	
III	544(22.4)	405(20.9)	139(28.5)	
Unknown	70(2.9)	59(3.0)	11(2.3)	
Breast cancer subtypes				<.001
HR+/HER2+ (luminal B)	319(13.1)	261(13.5)	58(11.9)	
HR+/HER2− (luminal A)	951(39.2)	820(42.3)	131(26.8)	
HR−/HER2+ (HER2 enriched)	194(8.0)	165(8.5)	29(5.9)	
HR−/HER2− (tripled-negative)	466(19.2)	369(19.0)	97(19.9)	
Unknown	496(20.4)	323(16.7)	173(35.5)	
Surgical approaches				<.001
BCT	773(31.9)	689(35.6)	84(17.2)	
Mastectomy	1574(64.9)	1174(60.6)	400(82.0)	
No/others	79(3.3)	75(3.9)	4(0.8)	
Radiation therapy				.043
Yes	1288(53.1)	1009(52.1)	279(57.2)	
No/unknown	1138(46.9)	929(47.9)	209(42.8)	
Chemotherapy				<.001
Yes	1831(75.5)	1421(73.3)	410(84.0)	
No/unknown	595(24.5)	517(26.7)	78(16.0)	

Abbreviations: BCT, breast-conserving therapy; HER2, human epidermal growth factor receptor 2; HR, hormone receptor.

**Table 2.** Comparison of clinicopathological characteristics between sentinel lymph node biopsy (SLNB) and axillary lymph node dissection (ALND) groups after propensity score matching (PSM).

Characteristics	Patients, no. (%)			*P*-value
	All patients *N* = 864	SLNB group *N* = 432(50.0)	ALND group *N* = 432(50.0)	
Age, years				.832
<50	311(36.0)	154(35.6)	157(36.3)	
≥50	553(64.0)	278(64.4)	275(63.7)	
Race				.949
White	631(73.0)	314(72.7)	317(73.4)	
Black	134(15.5)	67(15.5)	67(15.5)	
Others/unknown	99(11.5)	51(11.8)	48(11.1)	
T				.752
T3	652(75.5)	324(75.0)	328(75.9)	
T4a-c	212(24.5)	108(25.0)	104(24.1)	
N				<.001
pN0	848(98.1)	432(100.0)	416(96.3)	
pN+	16(1.9)	0(0.0)	16(3.7)	
Stage				.988
I	5(0.6)	2(0.5)	3(0.7)	
II	620(71.8)	311(72.0)	309(71.5)	
III	233(27.0)	116(26.9)	117(27.1)	
Unknown	6(0.7)	3(0.7)	3(0.7)	
Breast cancer subtypes				1.000
HR+/HER2+ (luminal B)	102(11.8)	50(11.6)	52(12.0)	
HR+/HER2− (luminal A)	255(29.5)	128(29.6)	127(29.4)	
HR−/HER2+ (HER2 enriched)	49(5.7)	25(5.8)	24(5.6)	
HR−/HER2− (tripled-negative)	176(20.4)	88(20.4)	88(20.4)	
Unknown	282(32.6)	141(32.6)	141(32.6)	
Surgical approaches				.621
BCT	156(18.1)	82(19.0)	74(17.1)	
Mastectomy	698(80.8)	344(79.6)	354(81.9)	
No/others	10(1.2)	6(1.4)	4(0.9)	
Radiation therapy				.729
Yes	511(59.1)	258(59.7)	253(58.6)	
No/unknown	353(40.9)	174(40.3)	179(41.4)	
Chemotherapy				.850
Yes	732(84.7)	367(85.0)	365(84.5)	
No/unknown	132(15.3)	65(15.0)	67(15.5)	

Abbreviations: BCT, breast-conserving therapy; HER2, human epidermal growth factor receptor 2; HR, hormone receptor.

### Survival Outcomes Between SLNB and ALND Groups in All Matched Patients and Subgroups

**Figure 2.**
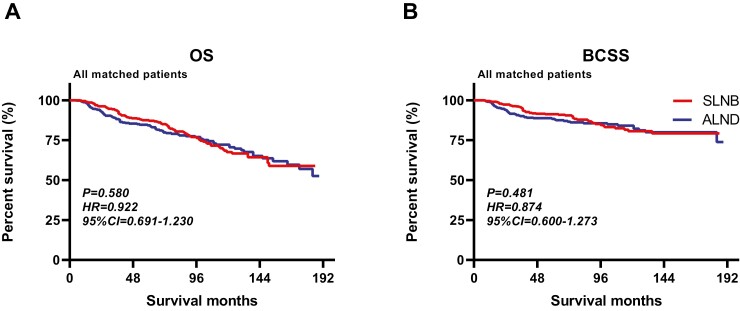
Kaplan-Meier survival curves of all matched patients. **A**. OS between SLNB and ALND groups; **B**. BCSS between SLNB and ALND groups. Abbreviations: ALND, axillary lymph node dissection; BCSS, breast cancer-specific survival; CI, confidence interval; HR, hazard ratio; OS, overall survival; SLNB, sentinel lymph node biopsy.

**Figure 3.**
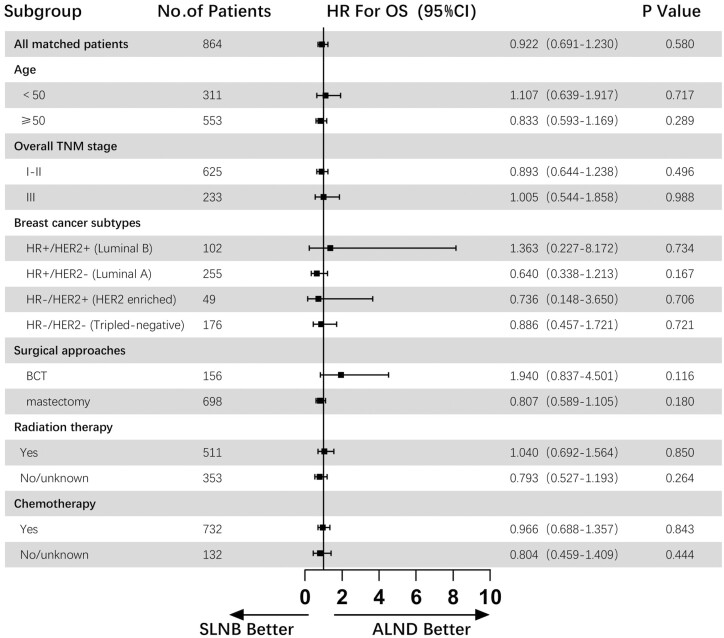
Forest plot of OS for patients in the entire cohort and subgroups. Abbreviations: ALND, axillary lymph node dissection; BCT, breast-conserving therapy ; CI, confidence interval; HER2, human epidermal growth factor receptor 2; HR, hazard ratio; HR, hormone receptor; OS, overall survival; SLNB, sentinel lymph node biopsy.

**Figure 4.**
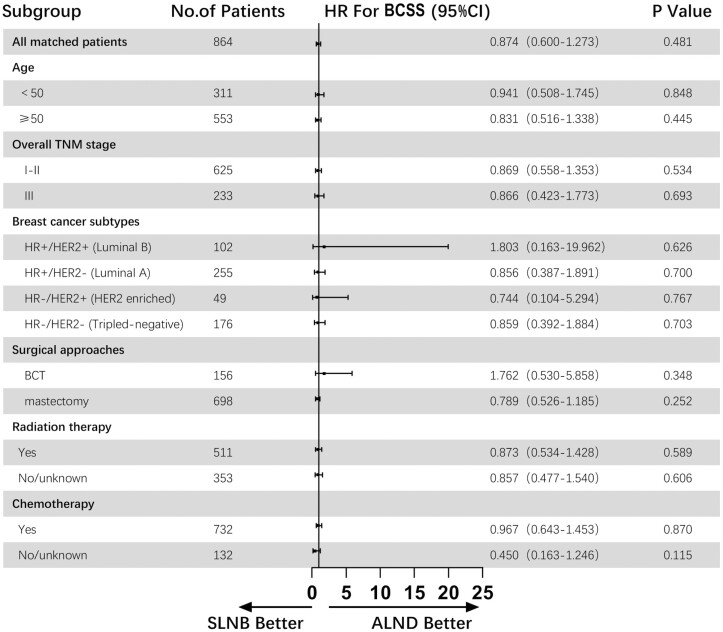
Forest plot of BCSS for patients in the entire cohort and subgroups. Abbreviations: ALND, axillary lymph node dissection; BCSS, breast cancer-specific survival; BCT, breast-conserving therapy; CI, confidence interval; HER2, human epidermal growth factor receptor 2; HR, hazard ratio; HR, hormone receptor; SLNB, sentinel lymph node biopsy.

With an average follow-up of 75 months, a total of 186 deaths were reported among 864 patients, 94 in the SLNB group and 92 in the ALND group. The OS and BCSS in the SLNB group were 78.2% and 87.5%, respectively, and that in the ALND group were 78.7% and 87.3%, respectively. The median number of regional lymph nodes examined in the SLNB group was 2, and that in the ALND group was 14. The Kaplan-Meier survival curves of OS and BCSS of the SLNB group compared with the ALND group in the matched population are presented in Fig. 2. And Figs. 3 and 4 show the forest plots of OS and BCSS for patients in the entire cohort and subgroups. According to the analysis results, among female patients with cN0, T3-4c, and M0 breast cancer, the SLNB group and the ALND group had similar OS (HR = 0.922, 95% CI, 0.691-1.230, *P* = .580) and BCSS (HR = 0.874, 95% CI, 0.600-1.273, *P* = .481), which remained significant in all the subgroup analyses. In addition, 3.7% of patients in the ALND group had pN+, and their overall mortality rate was not inferior to that of the total study population (12.5% vs. 21.5%).

### Survival Outcomes Among Patients With Different Molecular Subtypes Who Had Received Chemotherapy in the SLNB Group

There were no statistically significant differences in OS (*P* = .194) and BCSS (*P* = .303) among patients with different subtypes who had received chemotherapy in the SLNB group (Supplementary Fig. S1). Pairwise comparisons were made among different subtypes, and no significant differences in OS or BCSS were found between any 2 subtypes (Supplementary Table S1).

## Discussion

Currently, SLNB is the standard axillary staging surgery for cN0 and T1-2 breast cancer patients. However, whether it can be performed on female patients with T3-4c tumors is unclear. To explore its feasibility among these patients, we designed and conducted this retrospective study using data from the SEER database. After PSM, we found that among 864 cN0, T3-4c, and M0 female breast cancer patients, the OS and BCSS had no significant difference between SLNB and ALND.

Complications associated with ALND, such as arm lymphedema, pain, numbness, weakness, sensory loss, and so on, bring much inconvenience to patient’s work and daily life. According to a study from Ververs et al,^7^ after an average follow-up of 4.7 years, among those who underwent ALND, more than 20% of them reported pain, numbness, or weakness in the affected arm, and 9% of patients reported severe edema. Besides, 37% of patients said they had to give up a hobby or sport because of these troublesome complications. Compared with ALND, the complications related to SLNB are significantly reduced and patient’s quality of life is improved.^6,8,9,20^ At the same time, the performances of SLNB are also satisfactory in terms of survival and regional control. For instance, after a mean follow-up of 96 months, the NSABP B-32 trial^2^ reported that there were no statistical differences in OS, disease-free survival (DFS), and the risk of local and regional recurrence between SLNB and ALND among 3986 sentinel node-negative patients. Another study conducted by Bilimoria et al^21^ also found similar 5-year survival and risk of axillary recurrence between SLNB and ALND in 2203 patients who had microscopic nodal metastases. Axillary local recurrence after SLNB, with or without ALND, was a rare event (0.27% vs. 0.24%, respectively, with a median follow-up of 31 months), according to the research results from Arpana et al.^22^ However, patients with T3-4c tumors included in the above studies^2,21,22^ are too few (1.5%, 6.2%, and 1.2% respectively) to support the administration of SLNB among them. Our study included T3-4c breast cancer patients only, and also found that there were no significantly different in OS and BCSS between SLNB and ALND after a mean follow-up of 75 months among 864 patients, which indicates that the application of SLNB among these patients may also be safe enough.

We found 2 studies that demonstrated the feasibility of SLNB in patients with T3/T4b breast cancer. One was a retrospective study conducted by de Oliveira et al,^11^ involving 73 patients with T3/T4b breast cancer. They found that the identification rate of SLNB was 100%, and 60.3% of the patients did not have macrometastasis in the sentinel lymph nodes. These patients did not undergo ALND and were followed up for an average of 45 months without ipsilateral axillary local recurrence. Furthermore, the authors also found that 32.5% of T3/T4b patients (140 of 431) had pathologically negative sentinel nodes. If these patients do not receive SLNB, they may suffer from overtreatment, namely ALND. The limitations of this study were that it was retrospective, used single-center data, and had too few patients. Second, using a prospective breast cancer database, Chung et al^12^ selected 41 breast cancer patients with tumors ≥5 cm who underwent levels I-II ALND after SLNB. The authors found that the SLNB had an accuracy rate of 98% and an FNR of 3%, and 24.4% of patients had tumor-negative sentinel nodes. However, a small study population is also one of the limitations of this study. Besides, none of these 2 studies reported survival results after SLNB. The current study, using data from the SEER database, found similar OS and BCSS between SLNB and ALND among 864 female patients with cN0, T3-4c, and M0 breast cancer after PSM. Compared with them, our study population is much larger and more representative. Furthermore, we compared survival outcomes rather than the risk of axillary recurrence, which is more convincing in explaining the safety of SLNB. On the whole, we have the same conclusion, namely supporting the application of SLNB in patients with larger (≥5 cm) or locally advanced breast cancer.

Previous studies had reported varying accuracy rates of SLNB in T3-4c breast cancer patients. The results reported by Chung et al^12^ were encouraging. While the identification rate and FNR found by Arjunan et al^23^ were 86.4% and 21.4%, respectively, which is considered to be unacceptable, because the FNRs reported by most of the previous literature were all less than 10%.^1,24-27^ The following reasons may help explain the poor accuracy of SLNB reported by the latter. First, among the 38 patients with sentinel lymph nodes identified, 57.9% of them had fewer than or equal to 2 sentinel nodes removed. However, studies had shown that the FNR decreases as the number of sentinel lymph nodes removed increases.^1,28-30^ Second, 40.1% of patients were treated with a single tracer (methylene blue or radioactive colloid), while the FNR is lower when dual tracers are used for SLNB.^23,28,30^ Third, 86.3% of the patients included in the latter study had cN1 disease before neoadjuvant chemotherapy, and the FNR of SLNB among these patients was more than 10%.^30,31^

The following methods may help improve the accuracy of SLNB in T3-4c patients. First, try to remove more sentinel lymph nodes. When no more than 3 sentinel lymph nodes are identified, the peripheral lymph node sampling method can be adopted.^29^ Second, it is best to administer SLNB before neoadjuvant chemotherapy to prevent its accuracy from being affected. For patients who had received neoadjuvant chemotherapy, we can use a new tracer of SLNB, superparamagnetic iron oxide (SPIO). Studies had reported a 100% identification rate of SLNB with SPIO for patients after neoadjuvant chemotherapy.^32,33^ Besides, compared to the radioisotope, it can significantly improve the median of retrieved sentinel lymph nodes (3 vs. 2, *P* < .0001), while radioisotope is considered the standard tracer of SLNB in breast cancer patients after neoadjuvant chemotherapy.^33^

The American College of Surgeons Oncology Group (ACOSOG) Z0011 trial found that for T1-2 breast cancer patients, when they have ≤2 pathologically positive sentinel lymph nodes, ALND may be unnecessary if they plan to receive breast-conserving surgery plus whole breast radiation therapy.^34^ Affected by this result, the application of ALND decreases year by year.^35^ Combining this trend with the significant advantages of SLNB, we should consider SLNB first rather than ALND even facing patients with T3-4c breast cancer. However, we have to admit that the evidence available is not enough to support its extensive application among these patients. Its feasibility still needs to be fully proved by more large-scale randomized controlled trials or rigorous meta-analyses in the future.

The limitations of this study are as follows. First, there is no information on the sentinel lymph node mapping in the SEER database, therefore, it has the possibility that some people may undergo level I ALND or sampling. In our study, patients with 5 or fewer lymph nodes removed were categorized as having undergone SLNB alone and patients with 10 or more were included in the ALND group. Although previous studies^15-18^ have been done this way, this is still a limitation. Second, the SEER database does not specify whether chemotherapy was conducted in a neoadjuvant setting or adjuvant. Considering that around 40% of SLNB patients had HER2-enriched or triple negative breast cancer, these could be neoadjuvant patients for whom axillary surgery was de-escalated, however, their prognosis is completely different from patients undergoing upfront surgery. Therefore, we selected all patients who had received chemotherapy from the SLNB group and compared their survival difference according to molecular subtypes and finally found no statistical difference in OS and BCSS among them. Our results are consistent with the NSABP B-18 and B-27 trials,^36^ indicating that there is no significant difference in survival between patients receiving neoadjuvant chemotherapy and patients receiving adjuvant chemotherapy. Third, the results of SLNB, as well as who underwent SLNB plus ALND are unknown, so we cannot calculate the accuracy rate and FNR of SLNB to prove its technical feasibility among T3-4c patients. However, a previous study^15^ used the SEER database to select patients comparable to the ACOSOG Z0011 trial’s study population^34^ and ended up with the same results as the latter. This shows that although it is impossible to calculate the technical results of SLNB among patients from the SEER database, the survival results of these patients after SLNB are reliable. Fourth, this was a retrospective study, so there are selection bias and data loss.

## Conclusion

Using data from the SEER database, we found that the OS and BCSS after SLNB were similar to ALND among female patients with cN0, T3-4c, and M0 breast cancer when pN0 was presented. Therefore, SLNB may be safely performed on these patients, and we may omit ALND safely when pN0 is presented. However, studies regarding its feasibility in T3-4c breast cancer patients are far from enough currently to support its usage in clinical practice. The gap in this field still needed to be filled up by more large-scale randomized controlled trials or rigorous meta-analyses in the future.

## Supplementary Material

oyad038_suppl_Supplementary_Figure_S1

oyad038_suppl_Supplementary_Table

oyad038_suppl_Supplementary_Figure_Leend

## Data Availability

The data underlying this article are available in the Surveillance, Epidemiology, and End Results (SEER) database, at www.seer.cancer.gov.
