# Supplementary figures and images for: Survival After Sentinel Lymph Node Biopsy Compared with Axillary Lymph Node Dissection for Female Patients with T3-4c Breast Cancer
Source: Oncologist. 2023 Mar 17;28(8):e591–9. doi: 10.1093/oncolo/oyad038 (PMC10400163; doi:10.1093/oncolo/oyad038)

**A****OS**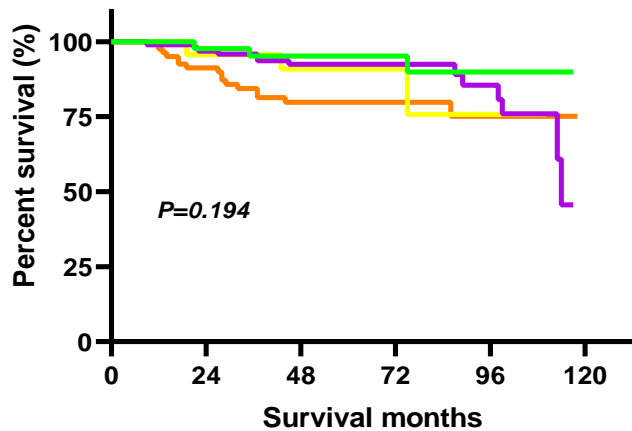**B****BCSS**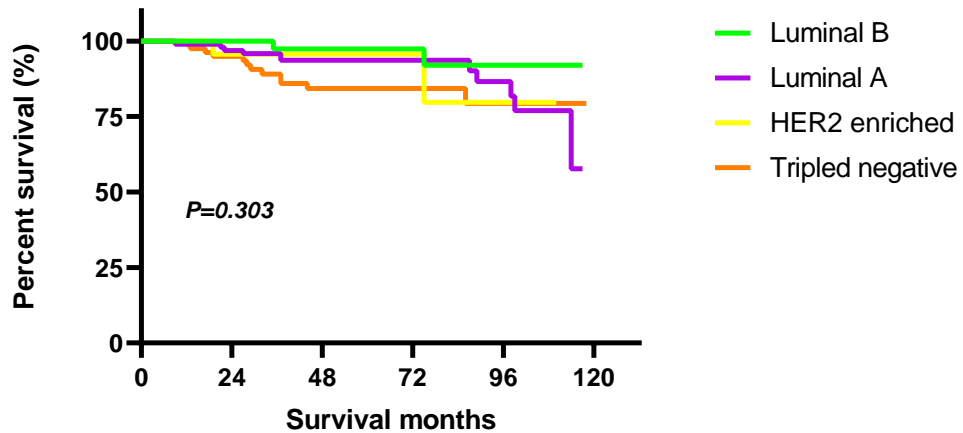

Supplement: oyad038_suppl_Supplementary_Figure_S1 [file oyad038_suppl_supplementary_figure_s1.pdf]
