# Supplementary material for: Survival After Sentinel Lymph Node Biopsy Compared with Axillary Lymph Node Dissection for Female Patients with T3-4c Breast Cancer
Source: Oncologist. 2023 Mar 17;28(8):e591–9. doi: 10.1093/oncolo/oyad038 (PMC10400163; doi:10.1093/oncolo/oyad038)
Supplement: oyad038_suppl_Supplementary_Table [file oyad038_suppl_supplementary_table.doc]

**Supplementary Table 1** Pairwise comparisons of log-rank on OS and BCSS among patients who had received chemotherapy in the SLNB group base on different molecular subtypes.

|  | P value^*^ | | | |
| --- | --- | --- | --- | --- |
| OS | Luminal B | Luminal A | HER2 enriched | Triple negative |
| Luminal B | / | 0.421 | 0.328 | 0.043 |
| Luminal A |  | / | 0.662 | 0.141 |
| HER2 enriched |  |  | / | 0.370 |
| Triple negative |  |  |  | / |
| BCSS |  |  |  |  |
| Luminal B | / | 0.310 | 0.381 | 0.060 |
| Luminal A |  | / | 0.997 | 0.293 |
| HER2 enriched |  |  | / | 0.370 |
| Triple negative |  |  |  | / |

* The P value is 0.008 after adjusting according to the number of pairwise comparisons between the four molecular subtypes, that is, P < 0.008 represents a statistical difference.

Abbreviations: OS, overall survival; BCSS, breast cancer-specific survival; SLNB, sentinel lymph node biopsy; HER2, human epidermal growth factor receptor 2.
