# Supplementary material for: Survival After Sentinel Lymph Node Biopsy Compared with Axillary Lymph Node Dissection for Female Patients with T3-4c Breast Cancer
Source: Oncologist. 2023 Mar 17;28(8):e591–9. doi: 10.1093/oncolo/oyad038 (PMC10400163; doi:10.1093/oncolo/oyad038)
Supplement: oyad038_suppl_Supplementary_Figure_Leend [file oyad038_suppl_supplementary_figure_leend.docx]

**Supplementary Figure 1.** Kaplan-Meier survival curves of patients who had received chemotherapy in the sentinel lymph node biopsy group. **(A):** OS among patients with different subtypes. **(B):** BCSS among patients with different subtypes.

Abbreviations: OS, overall survival; BCSS, breast cancer-specific survival; HER2, human epidermal growth factor receptor 2.
